# Epidemiological Survey on the Prevalence of Periodontitis and Diabetes Mellitus in Uyghur Adults from Rural Hotan Area in Xinjiang

**DOI:** 10.1155/2012/758921

**Published:** 2011-08-02

**Authors:** Gulinuer Awuti, Kurexi Younusi, Linlin Li, Halmurat Upur, Jun Ren

**Affiliations:** ^1^Department of Stomatology, the First Affiliated Hospital, Xinjiang Medical University, Xinjiang 830011, China; ^2^Xinjiang Medical University, Xinjiang 830011, China; ^3^College of Health Sciences, University of Wyoming, Laramie, WY 82071, USA

## Abstract

*Background and Aims*. This study was designed to explore the relationship between periodontitis and diabetes mellitus (DM) in Uygur adults from Xinjiang. *Methods and Results*. Data were obtained using questionnaire and oral examination. Participants (48.87 ±13.72 yr) were categorized into periodontitis and non-periodontitis groups in accordance with the chronic periodontitis diagnostic criteria. Based on gum inflammation, bleeding on probing, periodontal pocket depth and attachment loss, patients were further divided into mild, moderate and severe periodontitis groups. Among 962 subjects, 453 (47.1%) suffered from chronic periodontitis with a prevalence of type 2 DM and impaired fasting glucose of 9.5% and 11.4%, respectively. In the periodontitis group, the prevalence of type 2 DM was 75.6% compared with 22.4% in the non-periodontitis group. Likewise, the prevalence of impaired fasting glucose was 71.3% and 28.7% in periodontitis and non-periodontitis groups, respectively. The univariate logistic regression analysis revealed moderate and severe periodontitis as risk factors for DM (OR = 3.4, OR = 2.9). Multivariate logistic regression analysis showed that moderate periodontitis is independently associated with DM (OR = 4). *Conclusions*. Our data revealed that prevalence of DM is overtly higher in periodontitis patients than in individuals without periodontitis. Furthermore, moderate periodontitis is considered an independent risk factor for type 2 DM.

## 1. Introduction

Diabetes mellitus (DM), a chronic metabolic disease characterized by hyperglycemia, is often attributed to environmental and genetic factors. The prevalence of DM has risen dramatically in recent years, resulting in a rapid increase of diabetic patients. Asia in particular has the highest prevalence of diabetes in the world. Countries exhibiting the fastest rate in diabetic population growth include India and China, among many other developing countries [1]. Hyperglycemia triggers a wide variety of long-term complications in diabetics such as large vessel diseases, cardiomyopathy, and kidney and eye impairments [2, 3]. Periodontal diseases can be divided into gingivitis and periodontitis. Periodontitis is known as a chronic infectious disease of tissues surrounding the teeth which is induced by microorganisms. Periodontitis is a disease characterized by periodontal pocket formation, loss of connective tissue attachment, alveolar bone resorption, and gingival inflammation, ultimately resulting in tooth loss. When oral hygiene is compromised, oral bacteria may form a plaque biofilm, which is resistant to chemicals and immune cells [4, 5]. Without mechanical debridement, the plaque biofilm matures and causes gingivitis in a few days. Gingivitis represents chronic but reversible inflammation and can be usually treated by proper plaque control. Gingivitis typically extends to irreversible periodontitis for months or years [4–6]. Intriguingly, interaction and mutual influences between diabetes and periodontitis have been indicated [7, 8]. In fact, an ongoing longitudinal population-based observational study, the Hisayama study, reported a positive relationship between body mass index (BMI)/waist-hip ratio and the incidence of periodontal disease although neither impaired glucose tolerance nor diabetes was closely associated with the probing pocket depth [9]. Nonetheless, convincing evidence is still lacking on whether the therapeutic remedy for periodontal disease (such as antibiotic treatment) may achieve optimal glycemic control in diabetic patients [9]. Although preliminary study has been carried out on this important dental health issue [9], the precise underlying mechanisms remain elusive. To better understand the correlation between DM and periodontal disease, an epidemiological study was conducted in Uyghur adults from the town Cele in the Xinjiang Hetian region in 2010.

## 2. Methods

### 2.1. Study Subjects

The survey described here represents a cross-sectional study belonging to a sampling survey. Convenience sampling was used, and study subjects were recruited through television advertisements on the local television station. Inclusion criteria were as follows: willing citizens of Cele. The study protocol was approved by our institutional IRB committee, and written informed consent was obtained from all participants.

### 2.2. Study Protocols

#### 2.2.1. Questionnaire

The questionnaire was designed by an experienced research team. Following professional training, a group of Uyghur medical students fluent in the Chinese and Uyghur languages served as investigators and assisted in filling out the questionnaires. Contents of questionnaire include demographic information (gender, age), medical history (hypertension, diabetes, coronary heart disease, chronic renal disease, chronic respiratory diseases, etc.) behavior and personal habits (e.g., smoking), and family history. Results from the subsequent physical and blood tests were also included.

#### 2.2.2. Physical Examination

Participants were examined by trained medical students using uniform procedures and standards. Physical examination encompassed height, weight, waist circumference, abdominal/hip circumference, and blood pressure. Blood pressure was recorded three times, in accordance with the JNC 7 recommended standard [10]. Prior to each measurement, participants were asked to remain resting for at least 5 min.

#### 2.2.3. Collection of Blood Sample

Five mL fasting blood was collected from each participant. The samples were centrifuged, and the serum were placed in sodium fluoride tubes and kept frozen in individual containers. Serum sodium fluoride tubes were used to test the fasting blood glucose within 2 hours using a Johnson blood glucose meter. Serum was stored at −80°C in frozen containers in liquid nitrogen tanks.

#### 2.2.4. Oral Examination

According to the basic method issued by WHO oral health survey [11], gingival bleeding, probing depth (PD), and periodontal attachment loss (AL) were monitored using the plane mouth mirror, tweezers, and CPI periodontal probe, in conjunction with the probing method (probe's power <25 g) and visual examination. The mouth was divided into six sections, with index teeth 11, 16, 17, 26, 27, 31, 36, 37, 46, and 47 representing each section. Typically, testing results of index teeth represented the periodontal health for each section. All examinations were performed by an experienced dental specialist.

### 2.3. Diagnosis Criteria

#### 2.3.1. Diagnostic Criteria of Hyperglycemia

Diabetes mellitus (DM) and hyperglycemia diagnostic criteria are in line with the WHO standards issued in 1999 and American Diabetes Association standards [12, 13]. Impaired fasting glucose (IFG) was diagnosed with a fasting venous blood glucose level between 6.1 and 7.0 mmol/L and no history of DM. Hyperglycemia was diagnosed with a fasting venous blood glucose at 7.0 mmol/L or higher. A level of 7.0 mmol/L or above confirmed by repeated test at different days indicates the diagnosis of diabetes mellitus.

#### 2.3.2. Diagnosis of Periodontitis

Chronic periodontitis was categorized into the following categories. Mild periodontitis: gum inflammation and bleeding on probing, periodontal pocket depth ≤4 mm, and attachment loss of 1~2 mm. Moderate periodontitis: gingival inflammation and bleeding on probing, presence of pus, periodontal pocket depth ≤6 mm, attachment loss of 3~4 mm, and possible presence of slight loose teeth. Severe periodontitis: obvious inflammation or occurrence of periodontal abscess, periodontal pocket depth >6 mm, attachment loss ≥5 mm, and more than one loose tooth [14].

#### 2.3.3. Diagnosis of Metabolic Syndrome

Based on the 1999 WHO criteria [15], metabolic syndrome was diagnosed with at least 3 of the following components: (1) overweight and (or) obesity: BMI ≥ 30 Kg/m^2^; [16] (2) high blood glucose: FPG ≥ 6.1 mmol/L and (or) 2hPG ≥ 7.8 mmol/L, and (or) diagnosis of diabetes; [17] (3) hypertension: SBP/DBP ≥ 140/90 mmHg, and (or) diagnosis of high blood pressure and treatment of persons; (4) dyslipoidemia: fasting TG ≥ 1.7 mmol/L, and (or) fasting HDL-C < 0.9 mmol/L (male) or < 1.0 mmol/L (female).

#### 2.3.4. Diagnostic Criteria of Hypertension

Hypertension is defined as systolic blood pressure (SBP) ≥ 140 mmHg and (or) diastolic blood pressure (DBP) ≥ 90 mmHg, or taking hypotensors.

#### 2.3.5. Statistical Analysis

All data input is in duplicate using EpiData3.1 software with the logic and consistency checks. Data were analyzed using an SPSS software for Windows version 13.0. Measurement data was indicated with *x* ± *s*. Chi-square test was used to compare the percentage or count data. A logistic regression analysis was performed, using the forward Wald method with *α* = 0.05.

## 3. Results

### 3.1. General and Health Information

**Figure 1:**
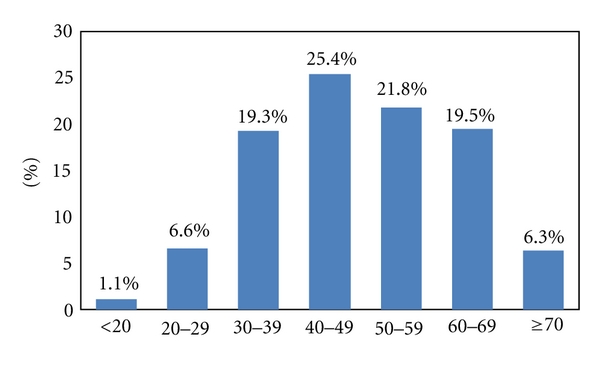
Age proportional ratio of the total sample population.

The Uyghur nationality in the town of Cele accounts for 95% of the total population of 140,000. The survey examined a total of 1099 cases of Uyghur adults. We received 1043 valid and complete questionnaires. 62 subjects failed to take the oral examination, and 19 cases lacked a full set of teeth; thus, 962 cases remained with complete data to be included. This study covered a population of 20 years old or older. The age proportional ratio of the total sample population is shown in Figure 1. The proportional ratio of different age groups is not the same, with the majority of the sample population represented by the 40~49 age group (25.4%). Sex ratio of the participants was male: female = 42.7%: 57.3%. General information of the survey is displayed in Table 1 (due to unfilled questionnaires, the number of effective response cases was not always consistent). General information about the periodontitis group and the non-periodontitis group is shown in Table 2. Table 2 indicates that in the elderly and male patients, the prevalence of impaired fasting glucose, diabetes, hyperlipidemia, metabolic syndrome, and hypertension is significantly higher in the periodontitis group than in the non-periodontitis group.

### 3.2. The Prevalence of Diabetes Mellitus and Periodontitis

In the survey, the prevalence of periodontitis was 47.1% (453 cases) in 962 individuals. The prevalence of mild, moderate, and severe periodontitis was 28.9% (278 cases), 10.2% (98 cases), and 8.0% (77 cases), respectively. The oral health conditions of the surveyed population are shown in Table 3. In the survey, prevalence of diabetes 9.0% (99/1043) and prevalence of impaired fasting glucose was 11.4% (101/880). The prevalence of DM in the periodontitis group was significantly higher than in the non-periodontitis group (75.6% versus 22.4%, *χ* ^2^ = 32.300, *P* = .000). Furthermore, the prevalence of impaired fasting blood glucose in individuals with periodontitis was significantly higher than in individuals without periodontitis (71.3% versus 28.7%, *χ* ^2^ = 25.322, *P* =  .000), (Table 2).

### 3.3. Regression Analysis of DM-Related Risk Factors

#### 3.3.1. Univariate Logistic Regression

A further logistic regression analysis was performed. The variables included age, gender, smoking, BMI, hyperlipidemia, hypertension, metabolic syndrome, and periodontitis. Classification valuation of independent variables is shown in Table 4. Results of the logistic regression analysis of DM risk factors are shown in Table 5. Our data revealed that age, BMI, hyperlipidemia, hypertension, metabolic syndrome, moderate periodontitis, and severe periodontitis are major risk factors to this group, indicating a likely correlation between DM and the above-mentioned risk factors. Without considering the other risk factors, the risks of moderate or severe periodontitis patients with diabetes were 2.9- or 3.4-fold higher than the nonmoderate or nonsevere periodontitis patients.

#### 3.3.2. Multivariate Logistic Regression Analysis

Using the independent variables of age, BMI, hyperlipidemia, hypertension, metabolic syndrome, moderate periodontitis and severe periodontitis, and diabetes as the dependent variable, a multivariate logistic regression analysis was performed using the forward Wald method with the thresholds of 0.05 for lead into and reject. Results of logistic regression analysis of DM risk factors are shown in Table 6. Our findings indicated that age, BMI, metabolic syndrome, and moderate periodontitis and severe periodontitis may serve as independent risk factors for diabetes. The risk of the moderate periodontitis patients with diabetes was 4-fold higher than those patients without moderate periodontitis.

## 4. Discussion

DM and periodontitis are common multigenetic and multifactorial chronic diseases with a higher incidence at increased age. Both of the morbidities negatively affect periodontal health and systemic health, thus affecting the quality of life [18]. An abundance of recent evidence has consolidated a bidirectional correlation between diabetes and periodontitis. While diabetes is an independent risk factor for periodontitis [19], periodontitis as a chronic inflammation has a negative impact on the metabolic control of diabetes [20]. In particular, periodontitis ranks sixth among all complications of diabetes [21]. 

The majority (76%) of Uyghur popultion from Xinjiang region reside around the Taklimakan Desert Oasis, among which 70% of the residents live in rural areas. Cele, located at the Southern edge of Taklimakan Desert, is a typical Uyghur rural area. Epidemiological data of periodontitis from adults living in the Hotan region display a much higher prevalence of periodontitis in Uyghur adults than the average among all age groups, according to the second national oral health epidemiological survey [22] and the United States NHANES1999~2000 [23]. Interestingly, the Uyghur adults are a high-risk group for periodontitis in the Hotan region. The current survey revealed a 47.8% prevalence of periodontitis in rural Uyghur adults in Cele, probably due to their unique ethnic lifestyle, oral hygiene habits, and economic conditions in the region. A survey for diabetes was performed in Tianjin regions, reporting a 51.41% prevalence of diabetes in patients with periodontitis (*χ* ^2^ = 7.363, *P* = .007) and a 27.68% prevalence of severe periodontitis (*χ* ^2^ = 4.967, *P* = .033) [9]. The data showed that the prevalence of diabetes patients with periodontitis was 75.6% (*χ* ^2^ = 32.300, *P* = .000), much higher than in the Tianjin study. Nonetheless, the prevalence of severe periodontitis (8.0%) was significantly lower than in the Tianjin study (27.68%).

In this survey, the overall prevalence of diabetes was 9.3%. The prevalence of diabetes in patients with periodontitis (17.7%) (453) was significantly higher than that of the non-periodontitis group (4.5%). In the periodontitis group with advanced age, impaired fasting glucose, hyperlipidemia, metabolic syndrome, hypertension, and other features, compared with the non-periodontitis group, there was a statistically significant difference. The prevalence of impaired fasting glucose, high blood lipids, metabolic syndrome, and hypertension was significantly higher than the non-periodontitis group. With the univariate logistic regression analysis for diabetes mellitus, and the related risk factors, our data depicted that age, BMI, hyperlipidemia, hypertension, metabolic syndrome, and moderate-to-severe periodontitis were risk factors for DM in the survey group. Notably, the univariate analysis showed that moderate to severe periodontitis was a risk factor for diabetes. The risks of moderate or severe periodontitis patients with diabetes were 2.4- or 1.9-fold greater than the nonmoderate or severe periodontitis patients. Given that multiple risk factors may affect the population prevalence of DM, our finding indicated that moderate-to-severe periodontitis was a risk factor in the survey group through multivariate regression analysis. The risks of moderate or severe periodontitis patients with diabetes were 3.0- or 1.3-fold greater than the patients without moderate or severe periodontitis. 

Ample evidence has suggested that periodontitis may lead to cardiovascular disease through bacteremia of periodontal pathogens and the corresponding antigen-mediated chronic inflammation or immune response [24, 25]. Meanwhile, inflammation is known to promote the onset and development of insulin resistance [26–29] and, subsequently, type 2 diabetes. Periodontitis can easily turn periodontal tissue into a proinflammatory environment through increased levels of inflammatory mediators. The accumulated proinflammatory mediators play a pivotal role in reducing the sensitivity of insulin signaling and glucose metabolism [30–34]. Inflammatory cytokines such as TNF-*α* and IL-6 are known to promote insulin resistance [35]. Challenge of adipocytes with proinflammatory cytokines such as TNF-*α* phosphorylates insulin-receptor substrate-1 (IRS-1) at Serine residue and impairs insulin receptor tyrosine kinase [33]. Uysal and colleagues reported that mice lacking TNF-*α* were resistant to obesity-induced insulin resistance [9]. Administration of IL-6 to otherwise healthy volunteers led to a dose-dependent increase in the fasting blood glucose [9]. These results suggest that inflammatory cytokines, which may promote both insulin resistance and chronic inflammatory diseases including periodontitis, are expected to augment insulin resistance and risk of cardiovascular diseases through production of proinflammatory cytokines within the lesion site. In addition, both TNF-*α* and IL-6 are produced in adipose tissues (e.g., one-third of circulating IL-6 is derived from adipose tissues) [9]. These lines of evidence suggest that obesity, diabetes, and chronic periodontitis are mutually related to one another. At this time, little evidence is available to confirm a solid and direct link between periodontitis and insulin resistance, apparently due to the lack of epidemiological and experimental evidence. Our study in Uygur adults has shown that Uyghur adults are a high risk group for periodontitis. Interestingly, the prevalence of diabetes in patients with periodontitis was much higher than the non-periodontitis groups. Our survey favors the notion that moderate and severe periodontitis should be considered independent risk factors for diabetes. Periodontitis displays a close relationship to diabetes mellitus in many ethnic groups [36, 37] although there is no direct epidemiologic evidence consolidating the positive correlation between diabetes and/or glucose intolerance and periodontal diseases. Further scrutiny is warranted with regard to the relationship between diabetes and periodontitis in large population scales.

## Figures and Tables

**Table 1:** General information of survey population.

Parameter	Effective response	Female (*n* = 598)	Male (*n* = 445)	Total (*n* = 1043)
Age	1043	47.24 ± 12.88	51.05 ± 14.50	48.87 ± 13.72
Smoking	1019	5	125	130
BMI	966	25.57 ± 4.40	26.19 ± 3.92	25.84 ± 4.21
Total cholesterol (mmol/L)	880	4.85 ± 1.80	4.86 ± 1.05	4.85 ± 1.53
Triglyceride (mmol/L)	880	1.86 ± 1.52	2.10 ± 1.62	1.96 ± 1.57
HDL-C (mmol/L)	879	1.30 ± 0.40	1.31 ± 0.57	1.30 ± 0.48
LDL-C (mmol/L)	877	1.97 ± 0.61	2.11 ± 0.59	2.03 ± 0.60
Mean SBP (mmHg)	970	118.72 ± 24.12	124.30 ± 21.02	121.10 ± 23.00
Mean DBP (mmHg)	970	75.56 ± 15.54	77.70 ± 14.46	76.59 ± 15.11
Fasting blood glucose (mmol/L)	880	5.06 ± 1.75	5.24 ± 2.53	5.14 ± 2.12
Metabolic syndrome	880	268	232	500
Hypertension	970	133	94	227
Diabetes mellitus	1043	48	51	99
Periodontitis	962	216	237	453

**Table 2:** General information of periodontitis group and the non-periodontitis group.

	Non-periodontitis		Periodontitis		*χ* ^2^	*P*
	*N* = 509	%	*N* = 453	%		
Age						
20–44	308	78.2	86	21.8	195.394	.000
45–59	146	44.4	183	55.6		
≥60	55	23.1	183	76.9		
Gender						
female	340	61.2	216	38.8	35.900	.000
male	169	41.6	237	58.4		
Smoking						
no	428	52.2	392	47.8	0.245	.620
yes	65	54.6	54	45.4		
BMI (Kg/m^2^)						
<25	219	52.4	199	47.6	0.160	.689
≥25	283	53.7	244	46.3		
Impaired fasting glucose						
no	420	55.3	339	44.7	25.322	.000
yes	29	28.7	72	71.3		
Diabetes						
no	487	55.8	385	44.2	32.300	.000
yes	22	22.4	68	75.6		
Hyperlipidemia						
no	234	57.9	170	42.1	9.961	.002
yes	215	47.1	241	52.9		
Metabolic syndrome						
no	227	61.0	145	39.0	20.403	.000
yes	222	45.5	266	54.5		
Hypertension						
no	414	55.6	330	44.4	9.854	.002
yes	95	43.6	123	56.4		

**Table 3:** Oral health conditions of survey populations.

Oral condition	Case	Ratio (%)
Normal	49	5.1
Gingivitis	460	47.8
Mild periodontitis	278	28.9
Moderate periodontitis	98	10.2
Severe periodontitis	77	8.0

Total	962	100.0

**Table 4:** Classification valuation of independent variables.

Variable number	Number
X1 (age)	18–39 years = 1, 40–59 years = 2, ≥60 years = 3
X2 (gender)	female = 0, male = 1
X3 (smoking)	no = 0, yes = 1
X4 (BMI)	BMI ≤ 25 = 0, BMI > 25 = 1
X5 (hyperlipidemia)	no = 0, yes = 1
X6 (hypertension)	no = 0, yes = 1
X7 (high blood glucose)	no = 0, yes = 1
X8 (metabolic syndrome)	no = 0, yes = 1
X9 (mild periodontitis)	no = 0, yes = 1
X10 (moderate periodontitis)	no = 0, yes = 1
X11 (severe periodontitis)	no = 0, yes = 1

**Table 5:** Univariate logistic regression analysis of DM risk factors.

	*B*	Wald	S.E.	OR	95.0% CI		*P*
					Lower	Upper	
Age	0.862	37.137	0.141	2.368	1.795	3.125	.000
Gender	−0.394	3.469	0.212	0.674	0.445	1.021	.063
Smoking	−0.186	0.368	0.306	1.204	0.661	2.193	.544
BMI	0.991	15.176	0.254	2.695	1.636	4.437	.000
Hyperlipidemia	0.767	9.722	0.246	2.152	1.329	3.485	.002
Metabolic syndrome	2.9	31.449	0.517	18.172	6.595	50.067	.000
Hypertension	0.494	4.280	0.239	1.639	1.026	2.619	.039
Mild periodontitis	0.241	1.054	0.235	1.273	0.803	2.019	.305
Moderate periodontitis	1.228	20.292	0.273	3.415	2.001	5.827	.000
Severe periodontitis	1.064	12.127	0.306	2.899	1.593	5.278	.000

**Table 6:** Multivariate logistic regression analysis of DM risk factors.

	*B*	Wald	S.E.	OR	95.0% CI		*P*
					Lower	Upper	
Age	0.474	6.136	0.191	1.607	1.104	2.338	.013
BMI	0.968	9.022	0.322	2.633	1.400	4.952	.003
Metabolic syndrome	4.874	57.121	0.645	130.903	36.98	463.375	.000
Moderate periodontitis	1.394	16.76	0.341	4.033	2.069	7.861	.000
Severe periodontitis	1.839	4.246	0.407	2.313	1.042	5.137	.039
